# Is this advertisement designed to appeal to you? Adolescents’ views about Instagram advertisements promoting ultra-processed products

**DOI:** 10.1017/S1368980024000533

**Published:** 2024-03-07

**Authors:** Gastón Ares, Lucía Antúnez, Florencia Alcaire, Virginia Natero, Tobias Otterbring

**Affiliations:** 1 Sensometrics & Consumer Science, Instituto Polo Tecnológico de Pando, Facultad de Química, Universidad de la República, By Pass de Rutas 8 y 101 s/n, Pando, Montevideo, Uruguay; 2 Centro de Investigación Básica en Psicología, Facultad de Psicología, Universidad de la República, Tristán Narvaja 1674, Montevideo, Uruguay; 3 Escuela de Nutrición, Universidad de la República, Montevideo, Uruguay; 4 School of Business and Law, Department of Management, University of Agder, Universitetsveien 17, Kristiansand, Norway

**Keywords:** Adolescents, Adolescent targeted marketing, Digital food marketing, Content analysis, Social media marketing, Public health policy

## Abstract

**Objective:**

Examine the key elements that characterise social media advertisements targeted at adolescents by asking adolescents which features of Instagram ads promoting ultra-processed products make them designed to appeal to adolescents.

**Design:**

Instagram ads promoting ultra-processed products and brands were selected from a database in which ads had been classified regarding whether they were primarily targeted at adolescents from an adult perspective. Adolescents completed a sorting task in small groups and were requested to reach a consensus through discussions and sticky notes regarding whether sixty ads were designed to appeal to them. The sorting task was analysed using content analysis based on inductive coding.

**Setting:**

One private secondary school and two after-school clubs.

**Participants:**

Convenience sample of 105 Uruguayan adolescents aged 11–17 years.

**Results:**

Ten categories were identified regarding the reasons for sorting ads as (not) designed to appeal to adolescents: product type, graphic design, explicit references to age groups, language, activities or themes, memes, celebrities, characters, promotions and novelty. Product type emerged as a key element, with adolescents perceiving ads as designed to appeal to them simply because they promoted specific products.

**Conclusions:**

This research contributes to the validation of criteria defined in previous studies and can be used for the development of tools to monitor the prevalence and power of adolescent-targeted digital marketing. However, the importance attributed to type of product suggests that regulations should not exclusively focus on exposure to digital marketing specifically targeted at adolescents but also on exposure to marketing in general.

Digital media have become a key part of the daily life of adolescents^(1)^, with such media content exposing them to marketing of ultra-processed products and fast food outlets across a wide range of platforms^(2–5)^. Adolescents are highly vulnerable to the persuasive effects of exposure to this type of marketing, although their cognitive skills are similar to those of adults^(6,7)^. Adolescence is characterised by high sensitivity to reward, reduced inhibitory control and increased susceptibility to social pressure and symbolism associated with product and brand consumption^(6–8)^.

Although the evidence is still scarce, exposure to digital marketing of unhealthy foods and beverages has been associated with increased recall, choice and intake of the advertised products among adolescents^(9,10)^. These effects are expected to be followed by medium- and long-term effects in adolescents’ eating habits and health^(11,12)^. However, empirical evidence on these effects is still very limited. One of the few recent studies on the topic has shown that self-reported exposure to social media marketing featuring food products and brands was positively associated with consumption of non-core foods^(12)^. This result matches the hierarchy of effects models of other types of food marketing reported for both adolescents and children^(9,13)^.

The persuasive effects of marketing does not only depend on exposing the target population to a given message but also on the power of the message itself, i.e. the creative content and the promotional techniques used to persuade^(14,15)^. Advertisements are expected to be more persuasive when their content is tailored to the interests and motivations of the target population^(16,17)^. Although adolescents are expected to be a specific target group of food marketing, few studies have explored the prevalence and power of food marketing targeted specifically at adolescents^(4,18–20)^. This type of research requires the definition of key elements that make advertisements particularly appealing to adolescents^(18)^.

A limited number of studies have defined indicators of adolescent targeted marketing^(4,18–20)^. Most indicators refer to elements of the advertisements that intend to capture adolescents’ interests and motivations (e.g. specific themes, activities, celebrities and products), or that adolescents identify with (e.g. adolescent language, adolescent models)^(4,18–20)^. The most frequent approach for the identification of these indicators has been reliance on researchers’ or experts’ opinions^(18)^. As far as can be ascertained, however, only three studies have engaged adolescents themselves to obtain their insights on the power of marketing; one involving outdoor advertisement and the other two digital marketing^(4,20,21)^.

In this context, the present study intended to contribute to the literature by expanding knowledge on the key elements that characterise social media advertisements targeted at adolescents through a participatory approach. Specifically, the aim of the present research was to identify adolescents’ views on the elements of Instagram ads promoting ultra-processed products that make them designed to appeal to adolescents. Active engagement of adolescents in the definition of criteria for the evaluation of the power of marketing has the potential to increase the external validity of research about the prevalence and power of food marketing targeted at adolescents. This approach is aligned with the UN Convention of the Rights of the Child. Article 12 establishes that children and adolescents have the right to be heard, especially regarding issues relevant to them, such as their health and well-being: ‘Children have the right to give their opinions freely on issues that affect them. Adults should listen and take children seriously’^(22)^.

## Materials and methods

A sorting task was used to address the research objective and explore adolescents’ criteria for regarding Instagram ads as (not) designed to appeal to them. This technique has been extensively used across several disciplines to study how people perceive objects^(23)^. It is based on asking participants to sort a series of objects and to identify the characteristics responsible for the perceived similarities and differences among them^(24)^.

### Participants

The study involved a convenience sample of Uruguayan adolescents. They were recruited at three institutions targeting adolescents from different socio-economic backgrounds: one private secondary school targeted at adolescents from medium/high socio-economic status and two after-school clubs targeting adolescents from low socio-economic status.

At each of the three institutions, all adolescents were invited to participate. Members of the institution staff sent an invitation letter to parents, who provided their informed consent to authorise their child’s involvement in the study. Adolescents authorised by their parents were invited to participate. Those interested in taking part in the study were asked to provide written informed assent. After analysing data from the three institutions, no additional data collection was deemed necessary as saturation was reached on the criteria underlying the classification of advertisements as (not) designed to appeal to adolescents^(25)^. A total of 105 adolescents, with ages ranging from 11 to 17 years (*M* = 15·6, s d = 1·9), participated in the study. Their main socio-demographic characteristics are shown in Table 1.

**Table 1:** Characteristics of the participants (*n* 105)

Characteristic	Number of participants	Percentage of participants (%)
*Gender*		
Female Male Other	60 43 2	57 41 2
*Age*		
11–14 years old 15–17 years old	59 46	56 44
*Socio-economic status*		
Low Medium/high	29 76	28 72
*Social media use*		
At least one social media YouTube TikTok Instagram Twitch Facebook Snapchat	105 100 89 85 39 15 11	100 95 85 81 37 14 10

### Stimuli

Ads promoting specific ultra-processed products or brands of such products were used as stimuli. They were drawn from a database of 2,104 ads generated by Instagram accounts of Uruguayan brands or branches of international companies between 15 August 2020, and 15 February 2021^(26)^. In a previous study, all the ads in the database had been classified regarding whether they were primarily targeted at adolescents or not from an adult perspective using a series of *a priori* indicators: references to adolescents or young adults; language or expressions used by adolescents; graphic design; memes; references to movies, TV or music; celebrities; references to videogames; references to high school or university and merchandising^(19)^. Ads were regarded as primarily targeted at adolescents if they included elements related to at least one of the indicators.

**Fig. 1:**
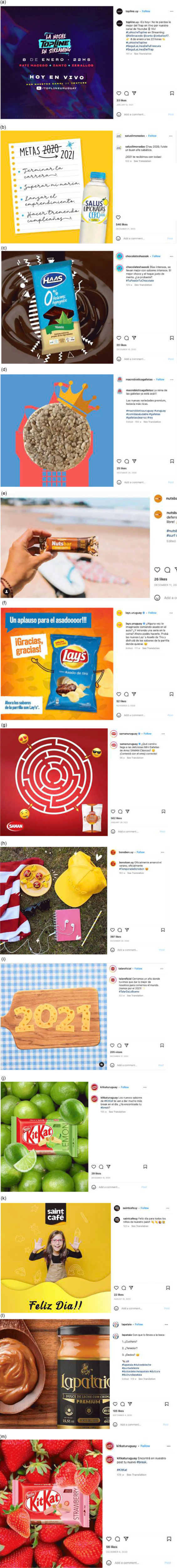
Examples of the Instagram ads used as stimuli in the sorting task: featuring at least one a priori indicator of marketing targeted at adolescents (a) ID 5, (b) ID 7, (c) ID 10, (d) ID 13, (e) ID 19, (f) ID 20, (g) ID 22, (h) ID 23, and not including any indicator (i) ID 32, (j) ID 35, (k) ID36, (l) ID 51, (m) ID 53 Translation of the ads to English: a) Topline streaming night. January 8th 10 PM. Fati Macedo + Zanto + Zeballos. Live today in our YouTube channel. Today. Don't miss the best of trap in our YouTube channel. #ToplineNight in streaming. b) 2021 goals. Graduate. Beat my mark. Launch my venture. Make a tremendous birthday party. Goodbye 2020, you were a good sabbatical year. 2021 we welcome you with the best vibes. c) 0% added sugar. Intense days are better with intense flavours. The best chocolate and just the right amount of mint. Have you tried it? #YourPassionYourChocolate. d) The queen of crackers is here. The new premium varieties are even tastier. e) This weekend make your defenses stronger in your outdoor activities. #nutsbar #healthylife. f) An applause for the cook! Thank you, thank you! Now grilled flavours are Lay's. Have you ever imagined yourself eating barbecue in your car? And watching a series in bed? Now you can do it. Try the new barbecue Lay's and enjoy the grilled flavours wherever you want. g) What path takes you to the delicious mini classic rice crackers? Answer with the right emoji. h) Summer has officially started. #BonoBonSeason. i) A year where we had to give the best of ourselves to conquer the world is coming to an end. To 2021! #TalarGivesYouTheBest. j) The new Kitkat flavours gonna give you more breaks during the da. Have you already find your #break? k) Happy day! Happy day to all the children in our country! l) How do you take it to your mouth? 1. Spoon? 2. Fork? 3. Fingers? m) Find in our post your new #break

Using the database, a total of sixty ads were randomly selected. Half of those ads (ID 1–30) were randomly selected from those that adult researchers a priori identified as primarily targeted at adolescents (*n* 371), whereas the other half (ID 31–60) were randomly selected from those ads that adults identified as not primarily targeted at them (*n* 1,733). The thirty selected ads identified as targeted as adolescents included elements related to all the *a priori* indicators of marketing targeted at adolescents, except for merchandising. Examples of the ads are included in Fig. 1(a) detailed description of the ads is included in the Supplementary Materials. Ads were printed in colour as small cards (12 cm × 8 cm).

### Data collection

The sessions were conducted with groups of 10–15 adolescents, in a quiet room in the institution where they were recruited. Participants were divided in small subgroups of 3–6 participants. A total of twenty-four subgroups were involved in the data collection, which differed in terms of their age and gender distribution.

After providing their informed assent, participants were handed the Instagram ads and were asked to complete a sorting task. They were instructed to look carefully at each of the ads and to discuss whether each of them were designed to appeal to adolescents or not. They were informed that they should reach an agreement to make the two groups of ads and to write down the reasons underlying the grouping using sticky notes. Care was taken to explain that the sorting task had no predetermined right or wrong answers. Three researchers were available in each of the sessions to monitor the groups’ work and to assist participants should they have any questions. After completing the task, participants were asked to fill out a form including questions about their gender, age and social media use. Participants completed the session in 15–30 min. Afterwards, they were debriefed about the aim of the task and a short group discussion about food marketing was held, after which participants received a brochure about healthy eating from the Uruguayan Ministry of Public Health.

### Data analysis

Upon completion of each session, the sticky notes detailing the reasons for sorting ads as (not) designed to appeal to adolescents were grouped and then transcribed intro a spreadsheet. Next, they were analysed using content analysis based on inductive coding. One of the researchers, with extensive experience in qualitative research and content analysis, coded the reasons for regarding an ad as (not) designed to appeal to adolescents into categories according to their meaning. A second researcher revised the proposed coding and suggested no changes. Examples of responses within each category were selected for illustrative purposes and translated to English.

A binary variable was created to indicate whether each of the ads was sorted as designed to appeal to adolescents or not in each of the subgroups. The percentage of subgroups regarding each of the ads as designed to appeal to adolescents was calculated.

## Results

### Criteria to sort ads as (not) designed to appeal to adolescents

Ten categories were identified in the content analysis regarding the reasons for sorting ads as (not) designed to appeal to adolescents (Table 2). All subgroups identified the promoted product as a key criterion considered in the sorting task. Participants stated that ads promoting foods liked and frequently consumed by adolescents, such as ‘junk food’, soft drinks, energy drinks, chocolate and snacks, were designed to appeal to them. On the contrary, ads promoting products they do not like or do not frequently consume were regarded as not designed to appeal to adolescents. Ads promoting culinary ingredients (e.g. tomato sauce), foods that require cooking (e.g. rice) or foods positioned as healthy (e.g. 0 % sugar, low calorie) were also regarded as not designed to appeal to adolescents.

**Table 2:** Categories identified in the content analysis of the reasons for sorting ads as (not) designed to appeal to adolescents. For each of the categories, a brief explanation of its content and examples of responses are provided

Category	Designed to appeal to adolescents	Not designed to appeal to adolescents
Type of product	Products liked and frequently consumed by adolescents *‘interesting products for our age’; ‘junk food and soft drinks that are more consumed by adolescents’; ‘delicious things’; ‘easy-to-get things that teens prefer to consume’; ‘chocolate’; ‘snacks’, ‘it makes you hungry’*	Products disliked or not frequently consumed *‘We don’t consume these products’; ‘food we dislike’; ‘teenagers are not interested in buying rice’; ‘we don’t cook’; ‘food for vegans and children’; ‘foods to eat at home and adolescents are rarely there’; ‘it isn’t tasty’; ‘light or healthy foods’, ‘it is for adults because they are rice crackers’*
Graphic design	Attractive graphic design ‘*attention grabbing images’; ‘modern posts’; ‘catchy colours’; ‘good combination of colours’; ‘different fonts’, ‘type of font’; ‘the design is adapted to adolescents’; ‘heart shape’*	Not attractive graphic design *‘not attention grabbing’; ‘colourless’* *‘dark’; ‘simple design’; ‘dull’; ‘too much text’; ‘too many objects on the image’; ‘the aesthetic is not for adolescents’; ‘emoji’*
Explicit references to age groups	Textual or visual references to adolescents *‘It shows adolescents*’	Textual or visual references to other age groups *‘it shows the hand of an old man’, ‘it is for children’; ‘talks about someone who has already graduated*’
Language	Words or expressions used by adolescents *‘adolescents’ words/expressions’; ‘short and catchy sentences’*	
Activities or themes	References to adolescent activities or themes *‘music’; ‘outdoor activities’; ‘it involves YouTube, streaming, everyday things for adolescents’; ‘a party for youngsters’; ‘it shows headphones and sunglasses’; ‘studies’, ‘Netflix’, ‘rugby’, ‘football’*	References to activities or themes not relevant to adolescents *‘for school’; ‘things adolescents are not interested in’; ‘not useful for us’; ‘it sets goals (for adults); ‘for the whole family’; ‘we’re not interested in the topic’; ‘the topics are not interesting’, ‘I don’t like rugby’*
Memes	Attention grabbing and funny memes *‘attention grabbing meme’; ‘funny’; ‘it’s a meme’, ‘they propose fun things’*	Memes regarded as not funny *‘I didn’t like the joke’; ‘I didn’t get the joke’;* *‘bad jokes’*
Celebrities	Celebrities attractive to adolescents ‘*Neymar*’	Celebrities not attractive to adolescents *‘teenagers don’t know or care about these people’, ‘I don’t know who she is’; ‘it shows a singer we don’t know’, ‘old football player’*
Characters	Cartoon characters relevant to adolescents *‘cartoon character’, ‘Frankenstein’; ‘Santa Claus’*	
Promotions	Promotions relevant to adolescents *‘you can win a car’; ‘it talks about price’*	Promotions not relevant to adolescents *‘the car is for grown ups’; ‘cars (for older than 18)’*
Novelty	Content regarded as new or innovative *‘innovative’; ‘new’*	Content regarded as not new or innovative ‘*They aren’t innovative*’

The graphic design of the ads was also identified as a key element underlying the sorting of the ads. Those sorted as designed to appeal to adolescents were described as attention grabbing, colourful and modern (Table 2). On the contrary, the design of ads sorted as not designed to appeal to adolescents were described as not attention grabbing, colourless, dark, simple, dull or having too much text.

Several categories related to adolescence emerged from adolescents’ accounts: explicit references to age groups, language and activities or themes (Table 2). The inclusion of explicit visual or textual references to adolescents, in contrast to other age groups, led participants to classify ads as designed to appeal to them. Another element of ads designed to appeal to adolescents was the use of language or expressions commonly used by them, as was the inclusion of references to adolescent activities or themes. In this sense, adolescents referred to music, parties, watching TV or series, outdoor activities, practicing sports or studying (Table 2). On the contrary, references to activities or themes not relevant to adolescents were identified as criteria for regarding ads as designed to appeal to other age groups. It is worth highlighting that all subgroups did not agree on how interesting some activities were for adolescents, such as rugby (Table 2).

Memes were identified as another tool to sort the Instagram ads, with attention grabbing and funny memes regarded as designed to appeal to adolescents. However, some of the subgroups indicated that certain memes were not funny or easily understandable and, consequently, were not effectively designed to appeal to adolescents (e.g. ID 28 and 29, see online supplementary material, Supplementary Material Table 1). Participants also identified the inclusion of references to some celebrities as a criterion to consider ads designed to appeal to adolescents, whereas references to unknown celebrities or those not relevant to adolescents themselves (e.g. a retired football player, described as old) were regarded as indicators of being designed to appeal to other age groups. The inclusion of cartoon characters was also mentioned as an element to appeal to adolescents.

Finally, some of the subgroups mentioned promotions and novelty as reasons to sort ads as (not) designed to appeal to adolescents (Table 2). Participants regarded ads including references to price promotions or raffles as designed to appeal to adolescents, as were ads regarded as innovative. Although some of the subgroups regarded an ad describing a car raffle as designed to appeal to adolescents, others regarded it as targeted as adult users, as drivers need to be 18 years old to obtain a driver’s license in Uruguay.

### Classification of the ads as (not) designed to appeal to adolescents

**Fig. 2:**
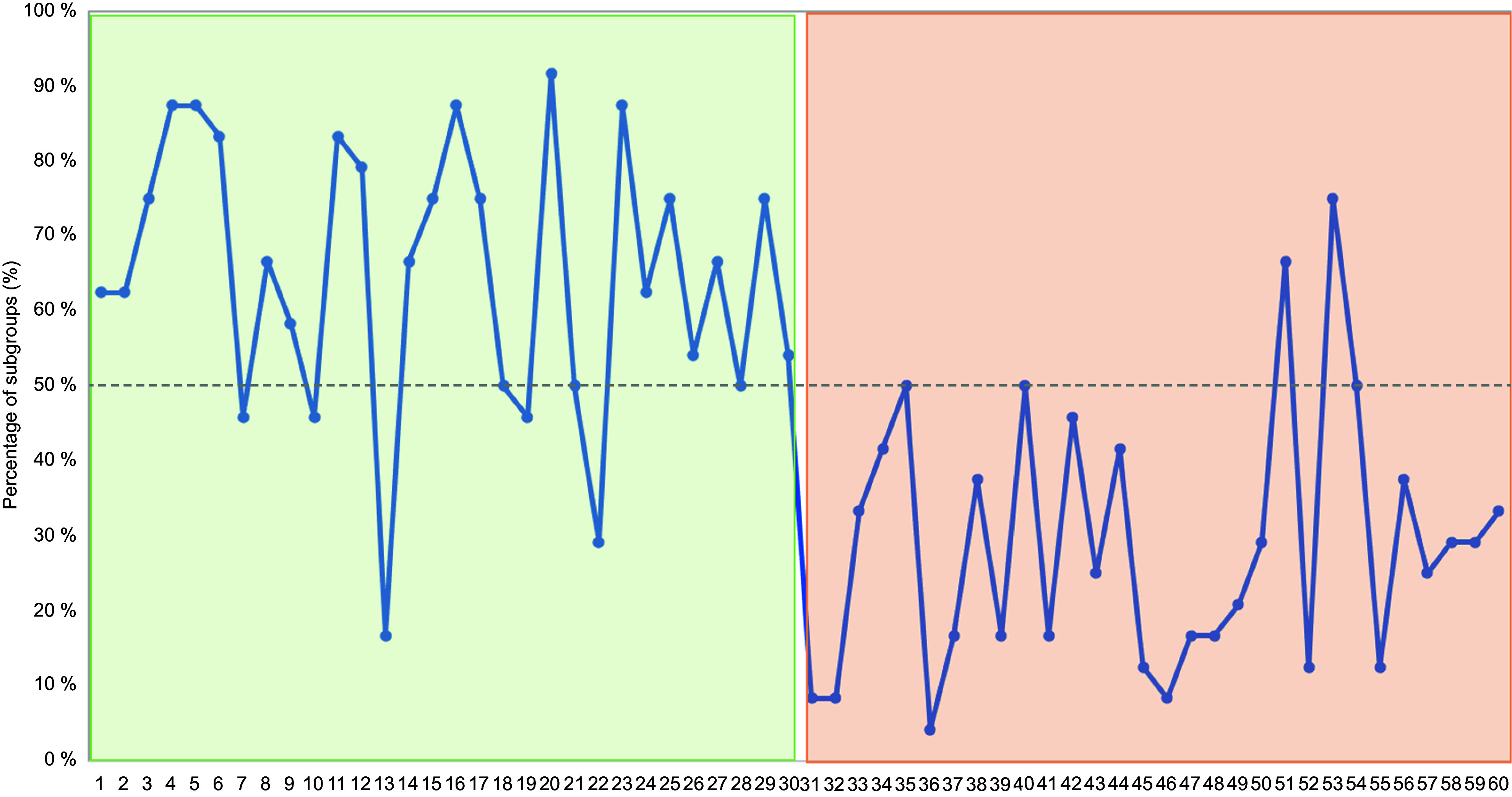
Percentage of subgroups of adolescents (*n* 24 subgroups, involving a total of 105 adolescents) who classified each of the Instagram ads as designed to appeal to adolescents. Ads from 1 to 30 (highlighted in green) included at least one a priori indicator of marketing targeted at adolescents, whereas ads from 31 to 60 did not include any indicator (highlighted in red)

Large differences were identified in the degree to which participants regarded the selected Instagram ads as designed to appeal to adolescents, as expected. The percentage of subgroups regarding ads as designed to appeal to adolescents ranged between 4 % and 92 % (Fig. 2). Ads including at least one of the a priori indicators of marketing targeted at adolescents (ID 1–30) were consistently classified as such by at least 50 % of the subgroups, except for five ads. On the contrary, ads not including any of the a priori indicators (ID 31–60) were not regarded as designed to appeal to adolescents by more than 50 % of the subgroups, except for two ads.

Three ads including elements related to the a priori indicators of marketing designed to appeal to adolescents were classified as such by less than 50 % of the subgroups, with these ads promoting products regarded as not interesting for adolescents: 0 % sugar chocolate (ID 10, Fig. 1(c)) and rice crackers (ID 13 and 22, Fig. 1(d and g), respectively). According to participants’ accounts, some of the reasons for not regarding the ad promoting flavoured water (ID 7, Fig. 1(b)) as designed to appeal to adolescents was the fact that it described setting goals, something adolescents do not normally do, and referred to graduation. Regarding ad ID19 (Fig. 1(e)), some of the subgroups indicated that the hand in the picture did not correspond to an adolescent.

Ads not featuring indicators of adolescent targeted marketing but still regarded as designed to appeal to this age group by more than 50 % of the subgroups typically promoted highly liked products: dulce de leche (ID 51, Fig. 1(l)) and strawberry flavoured chocolate (ID 53, Fig.1(m)). Although ads ID 53 (Fig. 1(m)) and ID 35 (Fig. 1(j)) promoted chocolate and shared a very similar design, they largely differed in the extent to which they were regarded as designed to appeal to adolescents (Fig. 2). Participants’ accounts identified product flavour as the key factor underlying the difference: they indicated that they did not find lemon flavour attractive (‘*we don’t like lemon’, ‘lemon kitkat*’).

## Discussion

Research on adolescents’ perspectives is necessary to inform the development of policies aimed at minimising the deleterious effects of exposure to digital marketing of unhealthy foods and beverages targeted at this age group^(27)^. The present study made a novel contribution to the literature by exploring adolescents’ views on the power of Instagram ads promoting ultra-processed products.

Results identified a wide range of elements that make Instagram ads perceived as being designed to appeal to adolescents. These elements were related to adolescent preferences, interests and motivations, in agreement with empirical evidence showing that the persuasiveness of advertisements is maximised when their content is tailored to the interests and motivations of the target population^(16,17)^. The relevance of these references matches adolescents’ need for belonging^(8)^.

The elements identified in the present research have been previously used in studies analysing the prevalence and content of marketing targeted at adolescents^(4,18,19)^. Therefore, results contribute to the validation of indicators of adolescent marketing. In this sense, results from adolescents’ classification of Instagram ads as (not) designed to appeal to adolescents demonstrate an adequate agreement with the categorisation performed in a previous study^(19)^.

Most of the indicators of adolescent targeted marketing identified in the present work require a subjective evaluation of the content of the ad. Participants referred to the appeal or relevance to adolescents when describing categories such as activities or themes, celebrities or promotions. This suggests that indicators of adolescent targeted marketing are not straightforward. Specific definitions for the indicators may contribute to reduce reliance on the subjectivity of researchers and policy makers when monitoring the prevalence and power of marketing targeted at adolescents. Adaptations of general definitions to the local context seem necessary to effectively capture ads attractive to adolescents, which may require active involvement of adolescents themselves. For example, adolescents’ views would be needed to identify personally relevant celebrities or themes.

Type of product emerged as a key element of the Instagram ads underlying the sorting task. Adolescents regarded ads promoting products they find appealing as designed to appeal to them, usually products with high content of sugar, fat and/or Na such as chocolate, soft drinks, energy drinks and savory snacks. On the contrary, ads promoting products they do not find appealing or positioned as healthy, were perceived as designed to appeal to other age groups. These results match the categories most frequently promoted to adolescents on Instagram^(19)^, as well as those identified as most prevalent in studies assessing adolescent exposure to digital food marketing^(3–5,28,29)^. Repeated exposure to marketing of unhealthy products using content related to adolescents may create social norms around the foods adolescents usually eat, reinforcing adolescents’ unhealthy dietary patterns^(30)^. Indeed, social norms have been recently reported to mediate the association between social media exposure and consumption of non-core foods^(12)^.

Promoting product categories frequently consumed by adolescents was a sufficient criterion to consider that an ad was designed to appeal to them. This suggests that adolescents may not only be attracted to advertisements that include specific elements to appeal to them, but to any other advertisements that catch their attention or promote products they like. A previous study assessing adolescents’ perceptions of outdoor advertising also pointed into this direction, as content not related to adolescents, such as food images and taste description, was identified as the most relevant aspect for making advertisements appealing^(21)^. The fact that adolescents regarded ads as designed to appeal to them only because they promoted specific product categories suggests the need to focus monitoring efforts on exposure to marketing in general rather exposure to marketing targeted at adolescents in particular.

Graphic design was another key criterion to classify Instagram ads as designed to appeal to adolescents, in agreement with results from previous qualitative studies involving adolescents^(2,4,20)^. Design elements making ads designed to appeal to adolescents included colours, brightness, text length, font, as well as overall aesthetics. In addition, references to celebrities relevant to adolescents were identified as another indicator of marketing designed to appeal to this age group. Celebrities and influencers have been shown to contribute to the memorability of advertisements and to increase their persuasiveness, particularly among children and adolescents^(2,5,31–33)^.

### Strengths and limitations

The key strength of the present research is its novelty and contribution to the field of food marketing. The involvement of adolescents, through a qualitative approach, ensures that valid and accurate information about their views of digital marketing was obtained. In particular, information about adolescents’ perception of the power of marketing was obtained, a topic few studies have addressed^(18,20)^. Results provide relevant insights to inform research on the prevalence and power of digital food marketing, as well as the design of public policies. Finally, the fact that the study was conducted in Uruguay, an emerging Latin American country, is another strength, as these populations have been underrepresented in the literature^(13)^.

Despite its strengths, the study is not free from limitations. First, a limited number of Instagram ads promoting ultra-processed products in Uruguay was considered. Although the ads were randomly selected from a database and included a wide range of products and content (see online supplementary material, Supplementary materials Table 1), additional elements not identified herein may emerge in further studies, thus meaning that the generalisability of the current findings should be interpreted with appropriate caution. Accordingly, further research is necessary to expand the results for the present work to other settings, types of products and social media platforms. Second, the qualitative nature of the study means that it is difficult to draw definite conclusions about the relative importance of different elements of the Instagram ads regarding the extent to which they were regarded as designed to appeal to adolescents. Therefore, additional quantitative research is needed to identify the effect of specific features of advertisements on their persuasiveness and appropriateness for different age groups. Such quantitative research has the potential to shed light on the mediators of the effect of digital marketing of unhealthy foods and beverages on adolescents’ attitudes and food choices. Finally, the current results were analysed at the aggregate level as data were collected through group discussions. This naturally precluded the evaluation of how individual characteristics, such as gender and age, might influence adolescents’ perceived power of the advertisements. Further research in this direction would make a relevant contribution to the literature.

### Conclusions

Through a participatory approach, the present work identified a series of indicators of adolescent targeted digital marketing. Results contribute to the validation of criteria defined in previous studies and can be used for the development of tools to monitor the prevalence and power of adolescent targeted digital marketing. However, adolescents identified some Instagram ads as designed to appeal to them only because they promoted products they like or frequently consume. This suggests that research and regulations should not exclusively focus on adolescents’ exposure to digital marketing targeted at them specifically. Instead, focus should be placed on exposure to marketing in general of all types of foods and beverages, regardless of whether such marketing material includes specific elements to appeal to adolescents or other age groups. From a regulatory perspective, a total ban of digital marketing of unhealthy foods and beverages, as proposed in the United Kingdom^(34)^, seems a promising way forward to protect adolescents.

## Supporting information

Ares et al. supplementary material Ares et al. supplementary material

## Supplementary material

For supplementary material accompanying this paper visit https://doi.org/10.1017/S1368980024000533.
