# Supplementary material for: Is this advertisement designed to appeal to you? Adolescents’ views about Instagram advertisements promoting ultra-processed products
Source: Public Health Nutr. 2024 Mar 7;27(1):e96. doi: 10.1017/S1368980024000533 (PMC10993065; doi:10.1017/S1368980024000533)
Supplement: Ares et al. supplementary material [file S1368980024000533sup001.pdf]

Supplementary material

Table 1. Description of the Instagram ads included in the study.

ID	Category	Image included in the ad	Text included in the ad	Identified as targeted at primarily adolescents	A priori Indicators of marketing targeted at adolescents identified in the ad (#)
1	Chewing gum	Old picture of a girl wearing a wedding dress with the following text: #Old meme. My friend: Ok, I don't want to get my hopes up too high. My friend five minutes later	Because we all have...🙄 Tag that friend who daydreams about marriage five minutes after meeting someone 👰🤵	Yes	iv
2	Chewing gum	Old picture of a girl washing clothes with the following text: #Old meme. Me at 8 behaving well to ask my mom a favor	Like if you suddenly wanted to wash the dishes or clean the whole house before they allowed you to go to that damn party 😂	Yes	iv
3	Corn snack	Drawing of a corn snack diving into a pool	The only sport we're willing to do everyday and that doesn't tire us. Running? What's that? Shall we eat?	Yes	ii, iii, iv
4	Potato chips	Video of football assists and goals	What assist did you enjoy the most? 🎁 Rafinha 🎁 Tadic 🎁 Carrasco 🎁 Mount 🎁 Square #UCL #UEFAChampionsLeague	Yes	vi
5	Chewing gum	Text on a blue background: T Topline streaming night. January 8th 10 PM. Fati Macedo + Zanto + Zeballos. Live today in our YouTube channel	Today. Don't miss the best of trap in our YouTube channel. #ToplineNight in streaming	Yes	v, vi
6	Cookies	Package of cookies on a colourful background with the text "choco choco choco chocolate chiqui chiqui chiqui chiquilin"	Do you know the Chiquilín cookie song? It starts this way: Choco Choco Choco Chocolate Chiqui Chiqui Chiqui Chiquilín The pleasure of eating... Do you remember how it goes?	Yes	iii
7	Flavoured water	Paper with the following text: ~~2020~~ 2021 goals. Graduate. Beat my mark. Launch my venture. Make a tremendous birthday party.	Goodbye 2020, you were a good sabbatical year. 2021 we welcome you with the best vibes	Yes	iii, viii
8	Dairy dessert	Vanilla dairy dessert with the text "*Danneteala!* Danette"	You open a Danette, stick the spoon in, start to savor it, and… 💥BOOM!💥 Explosion of flavor. You can't think of anything else anymore. Bye problems. Hello rainbow world 🌈🦄 So, when you need it, or just because you want... #Danettala with your favorite Danette 😎😋	Yes	ii

9	Energy drink	Teenagers playing rugby	Supporting (Instagram account) at all times 💪💪💪	Yes	i
10	Chocolate	Mint chocolate package on a melted chocolate background with white icons	0% added sugar. Intense days are better with intense flavours. The best chocolate and just the right amount of mint. Have you tried it? #YourPassionYourChocolate	Yes	iii
11	Ice-cream	Heart-shaped ice-cream on a pink background with the following text: "Love raffle. CrufiCupid"	Attention Crufilovers: in the month of love Crufi dresses up as Cupid! 💘 Follow us, upload a photo with your favorite Crufi ice cream, tag us and participate for an ice cream pack to enjoy with your crush on Valentine's Day 😍You have time until Friday the 12th to participate! Good luck 😉🙌🏻	Yes	iii
12	Flavoured water	Animated bottle with a speech bubble featuring the text "Hello summer!"	Celebrate the arrival of the long-awaited SUMMER with a Salus Lemonades. Cool off and enjoy, we deserve it. 😎🍋🌞	Yes	iii
13	Rice crackers	Picture of Rice cracker with a crown and a castle on the background	The queen of crackers is here. The new premium varieties are even tastier	Yes	iii
14	Dairy dessert	Package of a dairy dessert with the text: "You wished so hard it became true. New white chocolate Danette"	Drum roll please! 🥁🥁🥁🥁🥁 We present to you... 🎺CHAN-CHARARÁN🎺: THE NEW DANETTE WHITE CHOCOLATE FLAVOR! (you can clap in the comments). #Danette a new craving.	Yes	ii
15	Ice-cream	Ice-cream on a blue background with colorful flowers and the text "Mega offer Crufi Pop"	CRUFI POP but in its best version 🤩😉 Find them at an incredible price in all kiosks and stores!	Yes	iii
16	*Alfajor**	Alfajor on top of a laptop	Sunday afternoon plan: movies and our unique #AlfajorNegro #PuntaBallena ✌🏼 Is anyone else in it?	Yes	ii
17	Chocolate	Hand grabbing a chocolate, heart-shaped icons and the text: "You still haven't told him?"	The month of sweetness is coming to an end, and you still haven't told him what you feel? 💖 Say it in the sweetest way, say it with Bon o bon! Still haven't used our stickers? 😍	Yes	vii
18	Chocolate	Chocolates on a bright blue background	Chocolinas (cookies brand) and bon o bon, one heart! 🍫❤️	Yes	iii
19	Cereal bars	Hand grabbing a cereal bar	This weekend make your defenses stronger in your outdoor activities. #nutsbar #healthylife	Yes	i
20	Potato chips	Animated package of potato chips with the following dialogue: An applause for the	Have you ever imagined yourself eating barbecue in your car? And watching a series in bed? Now you can do it. Try the new	Yes	iii

		cook! Thank you, thank you! Now grilled flavours are Lay's	barbecue Lay's and enjoy the grilled flavours wherever you want		
21	Soda	Soda bottle on a green and yellow background with the text: "Being transparent is much better"	Because being transparent with yourself is also good 😎	Yes	ii
22	Rice crackers	Labyrinth with emoji on the entrance and a package of rice crackers on the exit	What path takes you to the delicious mini classic rice crackers? Answer with the right emoji	Yes	iii
23	Chocolate	Sunglasses, headphones, a notebook, a cap, a blanket, and a plate with chocolates on the grass. White star and heart-shaped icons on top.	Summer has officially started. #BonoBonSeason	Yes	i, iii
24	Fruit juice	Fruit juice bottle on a background showing a girl studying, featuring the text: "As you study Watt's 1 L"	The exquisite flavor of the fruit makes studying more bearable. 📚	Yes	i, viii
25	Chocolate	Drawing of Santa Claus on a kitchen catching fire	Christmas is coming…and Santa Claus needs a break! 🎄🍫 Have you tried the NEW KitKat Santa Claus? Pure chocolate and crunchy flavor	Yes	iii, iv
26	Chewing gum	Cellphone showing the following conversation: "- She lives with her parents, has two sisters, plays guitar, and has a dog. - How do you know? Haven't you told me that you didn't know her? - I stalked her buddy "	#Toxline: tag that friend who knows nothing but knows everything... 🔮🤫	Yes	ii, iii
27	Corn snack	A drawing of a lady looking like Frankenstein, featuring the text "Supermarket Frankenstein"	Today the heroine of the saga arrives. The quarantine caught her off guard and she used all available resources to go find Doritos at the supermarket 🧟‍♀️ What a quarantine monster were you?	Yes	ii, iv, v
28	Frozen pre-fried potatoes	Drawings of Instagram profiles of different types of French fried potatoes	Suggestions for today 🤩 *Not suitable for undecided people, we warned you 🙈	Yes	iv
29	Flavoured water	Bottle of flavoured water with black star-shaped icons showing a dictionary entry: Salus Lemonades 1 - Equivalent to touching cold water at the beach in an amazing summer day	To give you an idea, Salus Limonadas is the most refreshing thing you'll find this summer.	Yes	iv
30	Sausages	Picture of two Uruguayan football players dressed as medieval soldiers eating sausages	They changed history and continue to change it. 👏Three new flavors of Angus Sarubbi sausages! Caprese, cheese and olives, and the new Angus sausage with cheese and paprika. Try them!	Yes	iv

31	Rice	A package of rice and sushi rolls	Did you know? "Sushi" is a Japanese name that unites two words, "Su" (Vinegar) and "Shi-Meshi" (Rice). In other words, sushi could be translated as vinegared rice. 😲 Did you feel like making sushi? With Kansushi, it sure tastes better! 😉	No	-
32	Brand of dairy products	A drawing of a tablecloth and a cutting board with cheese arranged to show the number 2021	A year where we had to give the best of ourselves to conquer the world is coming to an end. To 2021! #TalarGivesYouTheBest	No	-
33	Yogurt	A package of yogurt, fruits, oat flakes, and a bowl of yogurt on a wood table, and the text "Eat what makes you feel good"	Because eating healthy means feeling good and there is nothing better than that feeling of well-being. Including fruits, cereals and dairy products every day is very important to be healthy and maintain balance. Have you tried this delicious combination with our light lactose-free yogurt yet?	No	-
34	Tomato sauce	A plate with spaguetti bolognese and a package of tomato sauce	Did you know that our Gourmet ® tomato sauces are ideal for preparing the meals you like the most? 🙂 Have you tried them yet? You will not regret it! 💥	No	-
35	Chocolate	A package of lemon flavoured chocolate on a background of lemons	The new Kitkat flavours gonna give you more breaks during the da. Have you already find your #break?	No	-
36	Breakfast cereals	A picture of a girl with the text "Happy day!" and the company name	Happy day to all the children in our country!	No	-
37	Tomato sauce	A bowl full of tomato sauce, next to a package of tomato sauce	Did you know that our Gourmet tomato sauce makes your meals more delicious? Choose Gourmet, and enjoy cooking with your family!	No	-
38	*Dulce de leche***	A plate with a waffle, dulce de leche and fruit, next to a package of dulce de leche	For a world of lactose-free products just as tasty as traditional ones. We promise that our lactose-free Dulce de Leche is more than delicious, you'll love it! It is perfect to eat with what you like best, with waffles, toast or cookies. What do you like to enjoy it with?	No	-
39	Chocolate	A lemon on a white and red package with the text: "A break to share"	You have no excuses to take a break, now they come with many more reasons to take one. #Break #KitKat	No	-
40	Energy drink	A can of an energy drink with the text "Leader"		No	-
41	Instant coffee	A coffee vending machine	Today more than ever we have the challenge of providing our customers with an experience that allows them to access products safely and conveniently. At Saint we	No	-

			have created an alliance between innovation and development with a new smart purchasing system that will drive our country towards excellence and responsible care. At Saint Hnos. we keep thinking of you!		
42	Fruit jam	A package of jam, surrounded by strawberries	We know what you can't miss with your morning coffee, some crunchy toast with our delicious jam! Made with patience and dedication 😍	No	-
43	Rice crackers	A guy in an office with a package of rice crackers and the text: "Do like Felipe. Imagine your Toyota Prius C!"	Felipe and his Toyota Prius C dance move invite you to participate in the SAMAN Promo 😂 Enter the code of any SAMAN product at promosaman.com.uy	No	-
44	Chocolate	The singer Julieta Venegas with the text "Julieta Venegas. November 7th 9PM. Live at Montevideo. Antel Arena" and the logo of the brand	Julieta Venegas arrives in Uruguay, and Bon o Bon allows you to be closer! How? Show that you are really a fan. 😍 Followed our account. 😍 Enter our stories. 😍 Complete the lyrics of the song. By doing so you are already participating in the raffle for tickets 😉	No	-
45	Fruit juice	A guy in an orange plantation with the text "At Citric we know each orange variety has its own harvest season"	We use 4 varieties of oranges to obtain the best 100% natural juice squeezed all year round, because we are #OrangeExperts	No	-
46	Instant coffee	A coffee plantation with the text: "Grown respectfully"	October 1st- International Coffee Day ☕At Nescafé and Nescafé Dolce Gusto we are committed to coffee-producing communities, focusing on sustainable production. We invite you to discover the dedication and care in each cup.	No	-
47	Fruit jam	A brown background with clouds and stars with the following text "Another year with you. To 2021, a year full of sweet moments"	We love accompanying you, like every year! Here's to a 2021 in which we continue to share good things. 💞	No	-
48	Frozen burger	A black background with a drawing of olive leaves	New Premium and Giant Sarubbi burgers. The greatest in the empire!	No	-
49	Sausages and cold cuts	Salami on a plate	Snack!	No	-
50	Packaged bread	A sandwich with the text "We go back to school and Bimbo has a packaged lunch full of tasty things for children"	Back to school with Bimbo! Nothing like a quick and delicious sandwich with our bread	No	-
51	*Dulce de leche***	A package and a spoonful of dulce de leche next to each other on a wood background	How do you take it to your mouth? 1. Spoon? 2. Fork? 3. Fingers?	No	-
52	Sausages and cold cuts	Plates full of cold cuts and sausages on a table	HAPPY 2021 TO ALL! The gatherings this summer are less numerous, but we can still	No	-

			indulge ourselves. Individual bites… why not? Let's enjoy responsibly.		
53	Chocolate	A package of strawberry flavoured chocolate on a background of strawberries	Find in our post your new #break	No	-
54	Ketchup and mayonnaise	Onion rings next to a bowl full of ketchup and a package of ketchup	Onion Rings Saturday! 🙋‍♂️ 🙋‍♂️ What can we eat them with? With our Gourmet Ketchup! #LetItBeGourmet	No	-
55	Brand of cold cuts	A natural cascade	The mouth of the Queguay Cascade is considered THE place in the world to get a Centenario Al Natural Ham 🌿🌎	No	-
56	Tomato sauce	Packages of tomato sauce on a background showing a plate of spaguetti bolognese on a table	Did you know that you can cook the tastiest meals with our variety of Gourmet sauces? 😉 Try different tastes and tell us which one you like best ✅ Make it Gourmet ®!	No	-
57	Packaged bread	A table with bread, fruit jam, a glass of juice and a package of sliced bread	We take care of you with our New Homemade Bread Sliced Zero Urupan. Ideal for a breakfast or light snack. Try it!	No	-
58	Cheddar dressing	A package of cheddar sauce next to a bowl of salad and bowl of corn snacks	The usual delicious flavor, now in a new super practical package! 🔝Try it!😉	No	-
59	Brand of *dulce de leche***	A beach with 2021 written in the sand	Today we are waiting for you until 18:30 pm Tomorrow 1/1 we celebrate with our families, and on 1/2 we will meet you at the usual times: 3:00 p.m. to 8:00 p.m.	No	-
60	Fruit juice	A drawing of a picnic and the text "Montevideo Pop Up. We're already here! We are waiting for you next to Linda Wall at Watt's stand"	This weekend come to @mvdpopup and we'll be waiting for you at the Kids section of Watt's with games and fruit juice. 📍 Lapataia Establishment, Punta del Este. 📅 Friday the 15th, Saturday the 16th and Sunday the 17th. 🕒 From 3 to 9 p.m. (Sunday from 3 to 8 p.m.).	No	-

Notes: (#) The following a priori indicators of adolescent targeted marketing were considered: i. references to adolescents or young adults; ii. language or expressions used by adolescents; iii. graphic design; iv. memes, v. references to movies, TV, or music; vi. celebrities; vii. references to videogames; viii. references to high school or university; ix. merchandising
